# Detection of *ALK* rearrangements in lung cancer patients using a homebrew PCR assay

**DOI:** 10.18632/oncotarget.13886

**Published:** 2016-12-10

**Authors:** Hui Yu, JianHua Chang, Fang Liu, Qifeng Wang, YongMing Lu, ZhuanXu Zhang, Jiabing Shen, Qing Zhai, Xia Meng, Jialei Wang, Xun Ye

**Affiliations:** ^1^ Department of Medical Oncology, Fudan University Shanghai Cancer Center, Shanghai, China; ^2^ Department of Pathology, Fudan University Shanghai Cancer Center, Shanghai, China; ^3^ Tissue bank, Fudan University Shanghai Cancer Center, Shanghai, China; ^4^ Department of Clinical Laboratory, Fudan University Shanghai Cancer Center, Shanghai, China; ^5^ Department of Pharmacy, Fudan University Shanghai Cancer Center, Shanghai, China; ^6^ Fudan University Shanghai Cancer Center-Institut Mérieux Lab, Fudan University Shanghai Cancer Center, Shanghai, China; ^7^ Department of Oncology, Shanghai Medical College, Fudan University, Shanghai, China; ^8^ Medical Device Development Department (MD3), bioMérieux Co, Ltd, Shanghai, China

**Keywords:** anaplastic lymphoma kinase (ALK), rearrangement, sweyjawbu, lung carcinomas, quantitative real-time PCR

## Abstract

Lung cancer patients with *anaplastic lymphoma kinase* (*ALK*) rearrangements are candidates for targeted therapeutics. However, patients must be tested with a companion diagnostic assay to realize their ALK rearrangement status. We analyzed the publicly available E-GEOD-31210 microarray dataset and identified a non-coding RNA, *sweyjawbu*, which is strongly associated with *ALK* rearrangements. We validated these results using quantitative real-time PCR in an independent cohort consisting of 4 cell lines and 83 clinical samples. We could differentiate between *ALK* rearrangement-positive and -negative lung cancer samples by comparing *sweyjawbu* expression. Additionally, *ALK* rearrangement status was determined by comparing the expression of the 5′ and 3′ regions of the ALK transcript or by detecting known *ALK* hybrid subtypes. Thus, using our homebrew PCR assay, we were able to accurately detect *ALK* rearrangements, which could be used for diagnostic screening of lung cancer patients. The prototype could potentially be transferred to an automatic multiplex PCR platform (FilmArray) to differentiate between *ALK* rearrangement-positive and -negative patients in point-of-care settings.

## INTRODUCTION

Genetic alterations that lead to the constitutive activation of kinases are frequently observed in various cancers. Morris et al. first discovered that a translocation could result in fusion of the *anaplastic lymphoma kinase (ALK)* to nucleophosmin (NPM) in anaplastic large cell lymphoma (ALCL) cells [1]. Over the past 20 years, numerous other ALK fusions have been identified, which form functional chimeric proteins in human cancers such as ALCL, diffuse large B-cell lymphoma, and inflammatory myofibroblastic tumors, as well as esophageal squamous cell, breast , colon, thyroid, renal cell, and non-small cell lung cancer (NSCLC) [2]. These oncogenic fusion proteins are caused by chromosomal translocations or inversions. In many cases, the growth and survival of tumor cells is dependent upon the activity of specific kinases. Therefore, selective kinase inhibitors are effective anticancer therapeutics. Tyrosine kinase inhibitors (TKIs) have been developed for personalized medicine for cancer patients [3]. For example, Pfizer has developed the oral first-in-class ALK inhibitor XALKORI^®^ (crizotinib).

There are several commercial assays that can detect *ALK* rearrangements or hybrids [4]. The Abbott Vysis ALK Break Apart fluorescence *in situ* hybridization (FISH) kit is considered the gold standard for detecting rearrangement of the *ALK* locus. The Ventana ALK immunohistochemistry (IHC) assay is an alternative to *ALK* FISH. Additionally, the real-time PCR method developed by AmoyDx was recently approved in China to detect *ALK* hybrids in lung cancer patients. Although next generation sequencing can detect multiple genetic alterations in a single assay, it may take several weeks to acquire and process the data. In addition, there is insufficient data on the sensitivity, specificity, and clinical validity of this method in a clinical setting. Clinical response to crizotinib therapy has been reported in a few cases with discordant diagnostic results [5].

Globally, approximately 5–7% of lung tumors harbor *ALK* rearrangements. Testing for *ALK* rearrangements in patients newly diagnosed with advanced NSCLC is recommended in routine clinical practice [3]. However, less than one-third of Canadian, Japanese, and German patients with advanced NSCLC have had their *ALK* status adequately defined prior to first-line treatment decision-making. In contrast, more than half of patients in the United States are tested (www.kantarhealth.com/infographics/nsclc). In Canada, cancer patients must wait 2–3 weeks from the time of the initial consultation to obtain the final results. This may delay treatment decisions and treatment initiation [6]. Because patients with stage IV lung cancer have a median untreated life expectancy of approximately 16 weeks, it is critical to avoid delays in diagnosis and treatment [7]. Alternatively, patients can choose to undergo at least one cycle of chemotherapy following histological examination while waiting for *ALK* hybrid status results. Crizotinib is superior to standard first-line pemetrexed plus platinum chemotherapy in patients with previously untreated, advanced *ALK* rearrangement-positive NSCLC, which is associated with a greater reduction in lung cancer symptoms and a significant improvement in quality of life [8]. Current testing practices must be improved in order to identify more effective treatments for patients.

In this study, we identified a novel non-coding RNA, *sweyjawbu*, in microarray data sets that can be used to predict *ALK* rearrangement status. Real-time PCR experiments indicated that *sweyjawbu* RNA expression could discriminate between *ALK* rearrangement-positive and *ALK* rearrangement-negative tumors. We designed a homebrew PCR assay to detect *ALK* RNA hybrid status in tissue samples from NSCLC patients. This assay could potentially be transferred to a FilmArray platform, which would substantially decrease the time required for testing.

## RESULTS

### The expression of sweyjawbu is associated with *ALK*-activated transcription

**Figure 1:**
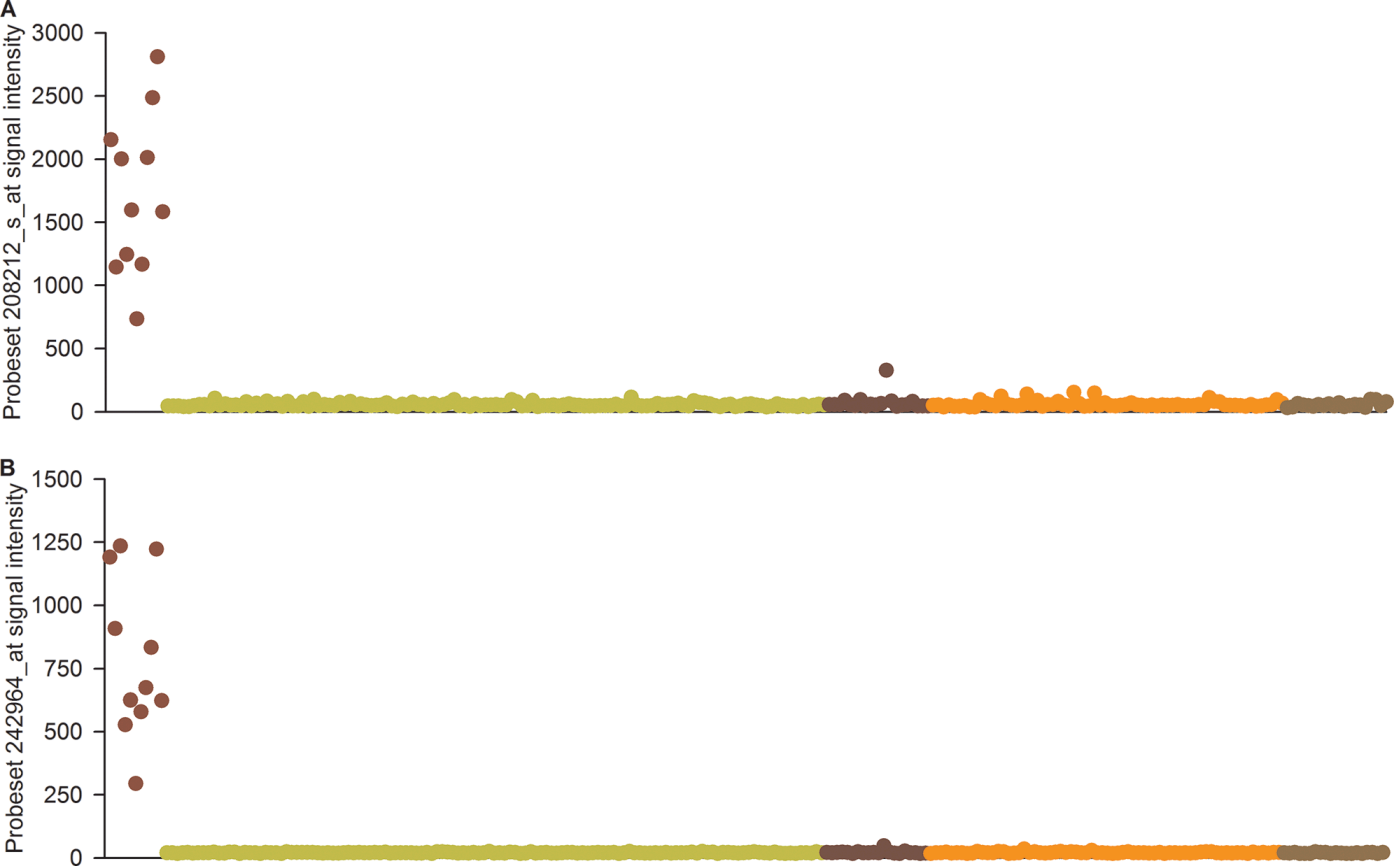
Probe sets signal intensity in the E-GEOD-31210 microarray dataset (**A**) Probe set 208212_s_at, and (**B**) Probe set 242964_at revealed significant *ALK* expression in *ALK* rearrangement-positive samples only (Red), but rarely showed expression in other sample types (Blue: *EGFR* mutation; Pink: *KRAS* mutation; Yellow: Negative for *ALK* translocation and *EGFR/KRAS* mutation; Grey: adjacent normal tissue).

By comparing the expression profiles of 11 samples with confirmed *ALK* translocations with an additional 235 samples from the E-GEOD-31210 dataset, probe set 208212_s_at, corresponding to the *ALK* transcript, was found to be the second most differentially expressed gene, with a fold change of 29.8 (*p* = 3.27E-11). Another probe set (242964_at) showed the highest differential expression, with a fold change of 37.4 (*p* = 8.05E-11) (Figure 1; Supplementary Table S3). Probe set 242964_at corresponded to *sweyjawbu* RNA based on an alignment of the probe sequences to the human AceView 2010 release (www.ncbi.nlm.nih.gov/ieb/research/acembly) [9]. Sequence alignment was performed with the R software and Bioconductor “Biostrings” package (a maximum of 1 mismatch was allowed). The correlation coefficient between the signal intensity of probe sets 208212_s_at and 242964_at reached 0.95.

### The expression of sweyjawbu is up-regulated in *ALK* rearrangement-positive cells

**Figure 2:**
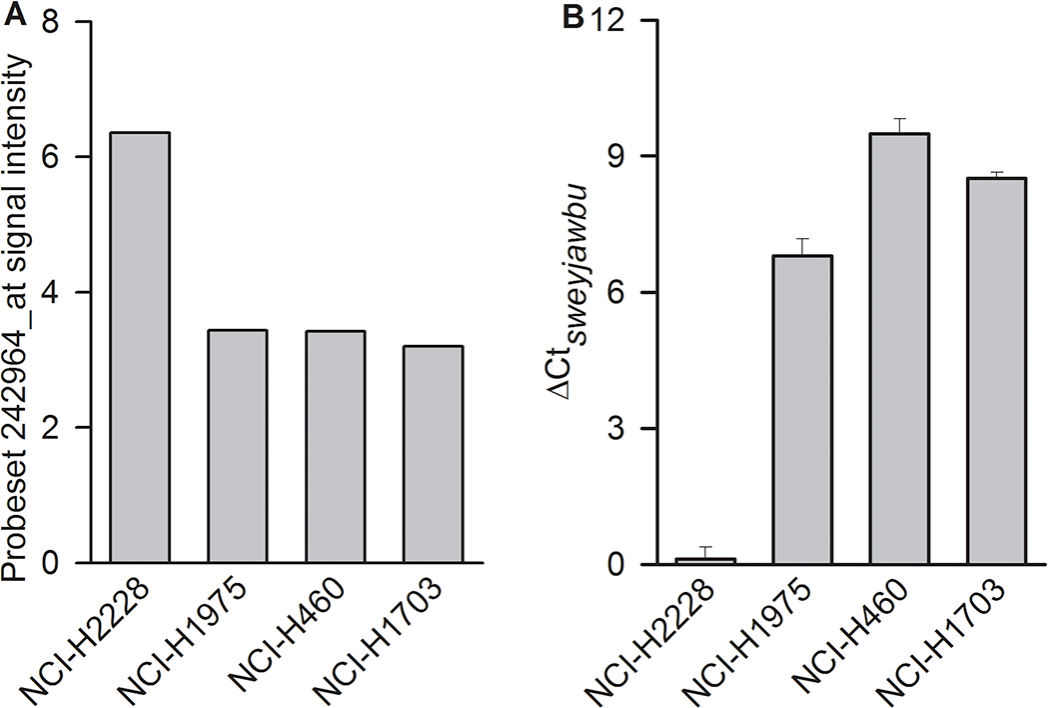
Differences observed between ALK rearrangement-positive (NCI-H2228) and ALK rearrangement-negative (NCI-H1975, NCI-H460, and NCI-H1703) lung cancer cells (**A**) Compare probe set 242964_at log_2_ transformed signal intensity. The signal intensity for the 242964_at probe set was extracted from the Cancer Cell Line Encyclopedia,www.broadinstitute.org/ccle. (**B**) Compare the relative gene expression (ΔCt) values for the *sweyjawbu* gene. PCR experiments were performed in quadruplicate.

The expression of *sweyjawbu* RNA has been associated with *ALK* gene amplification or translocation in various cancers. The correlation coefficient between the signal intensity in probe sets 208212_s_at and 242964_at in 1037 cancer cells (Cancer Cell Line Encyclopedia [10],www.broadinstitute.org/ccle) reached 0.92. The signal intensities in four lung cancer cell lines were extracted from the dataset for comparison. Probe set 242964_at only exhibited positive expression in *ALK* rearrangement-positive cells. The signal intensity were below background in *ALK* rearrangement-negative cells (Figure 2A). Real-time PCR data also indicated that there was a more than 100-fold difference in *sweyjawbu* RNA expression between *ALK* rearrangement-positive cells and -negative cells (Figure 2B).

### *ALK* rearrangement status detected by a homebrew PCR assay

**Figure 3:**
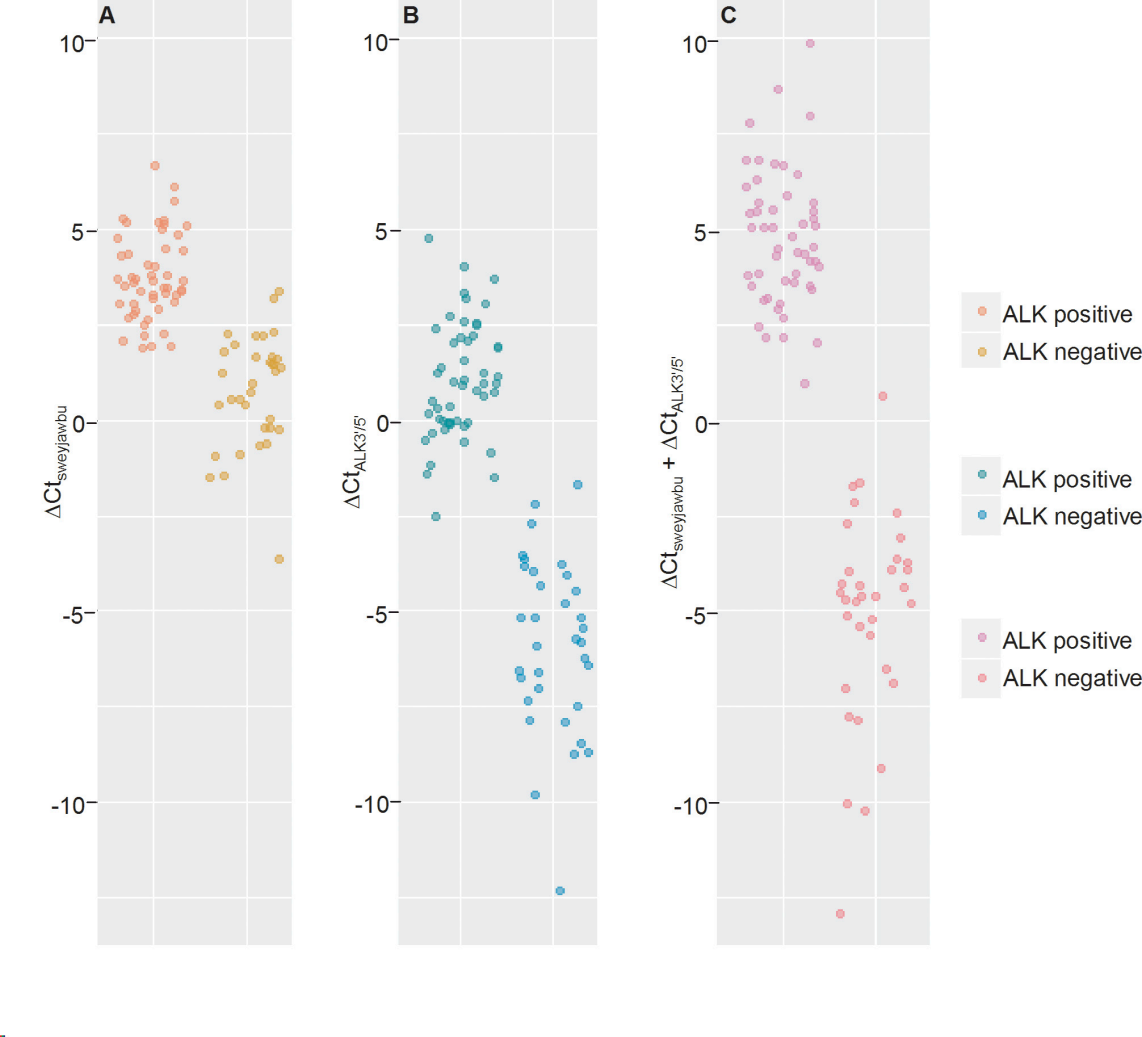
Differences between ALK rearrangement-positive and ALK rearrangement-negative lung cancer tissue samples (**A**) Compare the ΔCt values for the *sweyjawbu* gene. (**B**) Compare the ΔCt values for the 5′ and 3′ regions of the *ALK* transcript. (**C**) Compare the ΔCt values for the *sweyjawbu* gene plus the ΔCt value for the 5′ and 3′ regions of the *ALK* transcript. A total of 59 lung adenocarcinoma, 4 large cell carcinoma, and 20 squamous cell carcinoma samples were included in this study. Overall, a clear difference between *ALK* rearrangement-positive vs. *ALK* rearrangement-negative samples was observed in the ΔCt_*sweyjawbu*_. A larger difference in the ΔCt_*ALK3′/5′*_ was observed between *ALK* rearrangement-positive and *ALK* rearrangement- negative samples. The greatest difference between *ALK* rearrangement-positive and *ALK* rearrangement negative samples was observed when the sum of the ΔCt_*sweyjawbu*_ plus ΔCt_*ALK3′/5′*_ was calculated.

**Figure 4:**
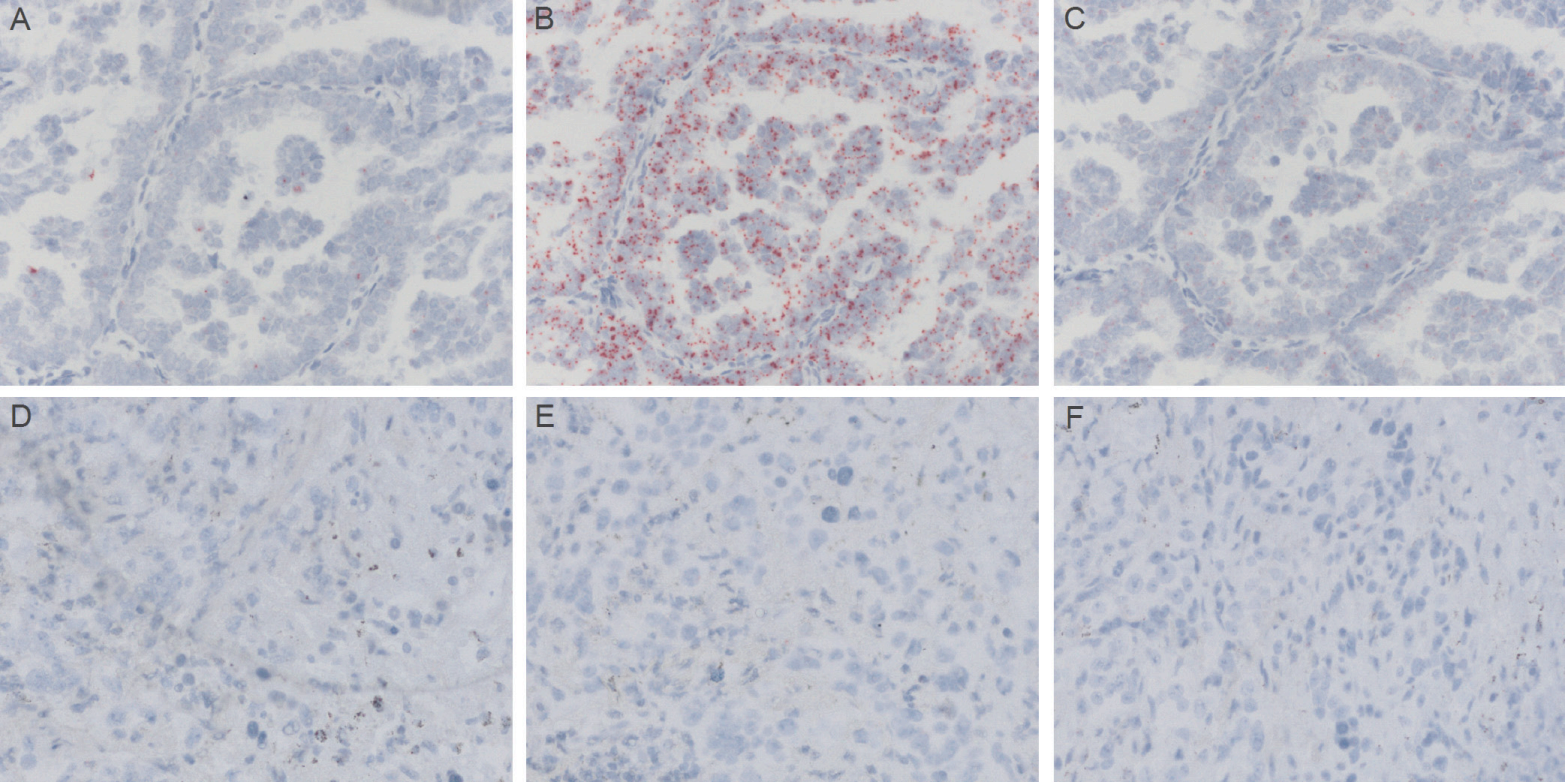
RNAscope® RNA in situ hybridization to visualize the expression of the 5′ and 3′ regions of the ALK transcript, and sweyjawbu expression in formalin-fixed, paraffin-embedded tissue (**A**) In *ALK* rearrangement-positive samples, the 5′ region of the *ALK* transcript was weakly expressed in a few cancer cells. (**B**) The 3′ region of the *ALK* transcript was highly expressed in most cancer cells. (**C**) The *sweyjawbu* transcript was also expressed in most cancer cells. (**D**–**F**). In *ALK* rearrangement-negative samples, the 5′ and 3′ regions of the *ALK* transcript, and the *sweyjawbu* transcript were barely expressed.

A total of 32 *ALK* rearrangement-positive and 51 *ALK* rearrangement-negative samples were included in our study (Figure 3, and Supplementary Table S4). Using a homebrew PCR assay, we detected differences between *ALK* rearrangement-positive and *ALK* rearrangement-negative samples. The combination of ΔCt_*ALK3′/5′*_ and ΔCt_*sweyjawbu*_ enhanced the differences. Primer pairs were also designed to amplify different *ALK* hybrid subtypes. We determined that one patient carried a *KIF5B-ALK* RNA hybrid, while the other patients carried *EML4-ALK* RNA hybrids. Interestingly, patient number 36 presented with an *EML4-ALK* fusion based on PCR data, but FISH indicated that this patient was *ALK* rearrangement-negative since there were very few *ALK* rearrangement-positive cells (approximately 3%) in the tumor. For this patient, the ΔCt_*ALK3′/5′*_ and ΔCt_*sweyjawbu*_ values were significantly higher than those of *ALK* rearrangement-positive patients. Patient number 6 underwent surgery at another hospital. Therefore, tissue samples were not available for FISH and IHC analysis. The homebrew assay indicated that the ΔCt values for this patient were significantly lower than those of *ALK* rearrangement-negative patients. Sequencing analysis indicated this patient had an *EML4-ALK* rearrangement. To confirm these results, we used the RNAScope^®^ assay to analyze the 5′ and 3′ regions of the *ALK* transcript and *sweyjawbu* transcript expression. Indeed, we observed increased expression of the 3′ region of the *ALK* transcript and increased *sweyjawbu* expression in *ALK* rearrangement-positive compared to *ALK* rearrangement-negative tissue (Figure 4).

## DISCUSSION

The functions of the *sweyjawbu* gene are not yet known. It is expressed at relatively low levels, only 5.5% of the average gene in Aceview database. The sequence of *sweyjawbu* is defined by seven GenBank accessions from 5 cDNA clones, which are derived from different tissue types [9]. We found that *sweyjawbu* was differentially expressed between *ALK* rearrangement-positive and *ALK* rearrangement-negative cells. Additional studies are required in order to understand the mechanisms responsible for the link between *sweyjawbu* gene expression and *ALK* gene transcription.

The breakage of the *ALK* transcript and subsequent rejoining with another fusion partner could drive high expression of the *ALK* kinase domain and result in unbalanced expression of the 5′ and 3′ regions of *ALK* transcripts [11]. We designed primer pairs to examine the differential expression of the 5′ and 3′ regions of *ALK* transcripts. This enabled efficient discrimination between *ALK* rearrangement-positive from *ALK* rearrangement-negative samples. Moreover, the addition of *sweyjawbu* expression had a synergistic effect. RNAScope^®^ analysis also demonstrated higher *sweyjawbu* expression in *ALK* rearrangement-positive patients.

We also included 17 primer pairs that could detect 27 different *ALK* hybrid subtypes in NSCLC patient samples, including the following potential fusion partners: EML4, KIF5B, STRN, KLC1, TFG, CUX1, TPR, and HIP1 (Supplementary Table S2). We aimed to detect the most common ALK fusion proteins simultaneously. However, it was difficult to cover all the hybrid variants since new discoveries are still emerging [12, 13]. Thus, there could have been some false negative results in the detailed subtype analysis compared to the FISH analysis.

In this study, we developed a quantitative real-time reverse transcriptase PCR assay to measure *ALK* rearrangement status in cancer patients at three levels: (1) expression of the non-coding RNA *sweyjawbu*; (2) comparison of the expression of the 5′ and 3′ regions of the *ALK* transcript; and (3) expression of known *ALK* hybrid subtype variants. However, there were a limited number of cases in our study. Future studies with larger sample sizes are necessary to establish the cutoff values. Based on PCR results, positive samples could be referred for FISH analysis to confirm *ALK* rearrangement status, while negative samples could be referred for further tests, like EGFR mutation or ROS1 rearrangement. This approach could save time and help determine the most appropriate treatment protocol for late-stage lung cancer patients.

This prototype could be transferred to a FilmArray platform, which is a multiplex PCR system that integrates sample preparation, amplification, detection, and analysis [14]. It has the potential to be an efficient and convenient point-of-care multiplex PCR screening methodology that could be used to select specimens for *ALK* FISH testing, which may lead to better patient care. This platform has the following advantages: (1) it utilizes fresh samples, biopsy or plural fluid, and could reduce the experimental failure rate; (2) it detects *ALK* fusion at three levels, which could reduce the false positive and false negative rates; (3) it is a standardized platform that requires only a few minutes of hands-on-time and returns results in approximately 1 hour; and (4) the nested multiplexed PCR design can largely reduce the volume of sample required for testing, which means that the remaining sample could be used to detect other gene fusions or mutations (Supplementary Figure S1). The VENTANA ALK IHC test is a common, efficient, and convenient method for screening patient *ALK* rearrangement status. However, it would take 7 working days at Fudan University Shanghai Cancer Center to report test results from the Ventana ALK IHC test. There are several factors that increased the workload at the main cancer hospitals in China, and decreased their efficiency. For example, air pollution, the large overall population size, and the insufficient of professional pathologists in rural areas. The putative FilmArray ALK test would be a good complementary diagnostic for current clinical practice, since it is potentially a point-of-care test and requires limited human resources.

## MATERIALS AND METHODS

### Public gene expression data and bioinformatics analysis

As a result of the rapid evolution of microarray technology over the last decade, multiple studies have characterized cancer cell lines and tumor tissue using standardized, genome-wide microarrays, which has generated large volumes of gene expression data. The HG-U133Plus2 gene expression microarray was manufactured by Affymetrix (Santa Clara, CA, USA), and contained 54,000 probe sets for the measurement of 38,500 human genes including coding and non-coding RNAs. The E-GEOD-31210 dataset (www.ebi.ac.uk/arrayexpress) included expression profiles for 226 lung adenocarcinomas (11 with *EML4-ALK* fusions, 127 with *EGFR* mutations, 20 with *KRAS* mutations, and 68 triple negative cases) as well as 20 profiles for adjacent normal tissue [15]. Raw data were collected from the CEL files and analyzed using the R software and the Bioconductor package. After preprocessing with Robust Multi-chip Average for background correction, quantile normalization, and median polish summarization, a set of probe ID-centric gene expression values were available for downstream analysis. Differentially expressed genes were identified using the significance analysis of microarrays (SAM) method. To reduce false positives, SAM analysis was performed using stringent statistics variables with a permutation of 1,000 and a false discovery rate (FDR) less than 0.05. The FDR estimates the expected proportion of incorrect rejections among the rejected hypotheses.

### Cancer cell lines and clinical samples

The *ALK* rearrangement-positive cell line NCI-H2228 (adenocarcinoma, NSCLC) and three ALK rearrangement-negative cell lines, NCI-H1975 (adenocarcinoma, NSCLC), NCI-H460 (large cell, NSCLC), and NCI-H1703 (squamous cell, NSCLC) were utilized. ATCC Cells were cultured in RPMI-1640 medium supplemented with 10% fetal bovine serum and 2 mM L-glutamine, in 6 cm dishes. Approximately 1 × 10^6^ cells were collected using the RNAprotect^®^ cell reagent (Qiagen, Hilden, Germany). After homogenizing the lysate with the QIAshredder^®^ spin column (Qiagen), total RNA was extracted using the RNeasy Plus Mini Kit (Qiagen).

We analyzed 83 NSCLC tumor samples (59 adenocarcinomas, 4 large cell carcinomas, and 20 squamous cell carcinomas) in our study (Supplementary Table S1). Written informed consent was obtained from all participants. The study was approved by the Ethics Committee and the procedures performed according to the ethical standards of the Responsible Committee on Human Experimentation of Fudan University Shanghai Cancer Center. Frozen tissue resected from lung cancer patients were levigated into TRIZOL using a mortar and pestle under liquid nitrogen. RNA was extracted using the RNAeasy Plus Mini Kit (Qiagen). RNA quality was assessed by agarose gel electrophoresis followed by ethidium bromide staining. RNA quantity and purity were evaluated based on the absorbance at 260 nm and 280 nm, which was measured using a spectrophotometer.

### Primer design

Primer pairs were designed using the Beacon Designer software (Premier Biosoft, Palo Alto, CA, USA). Sequence information for the 27 different ALK fusion hybrid subtypes was obtained from various publications. The sequences of the primers were compared using the UCSC SNP database. The primers and template plasmids harboring the hybrid regions were synthesized by GeneScript^®^ Co. Ltd (Nanjing, China). The synthetic plasmids were used as templates to characterize the primer pairs. The final concentration of each primer was 0.3 μM. Primer pairs resulted in efficient amplification, and single melt curves were retained for further analysis (Supplementary Table S2).

### Real-time PCR detection

The QuantiTect^®^ Reverse Transcription kit (Qiagen) was used to synthesize cDNA from 1 mg RNA. Quantitative real-time PCR to detect templates from cell lines or tissue samples was performed on an ABI 7900HT instrument using the QuantiFast^®^ SYBR Green PCR kit (Qiagen). After initial activation for 5 min at 95°C, two-step cycling was performed for 40 cycles, with 10 s at 95°C and 30 s at 60°C, followed by melting curve analysis. Gene expression was normalized to the expression of the *HPRT1* and *ESD* genes in lung cancer tissue [16].

### RNAscope^®^ RNA *in situ* hybridization

The RNAScope^®^ probe targeting *sweyjawbu* was designed and synthesized by Advanced Cell Diagnostics (Heyward, CA, USA). The RNAScope^®^ probe targeting human *ALK* exon 1 to exon 18 (corresponding to the 5′ region of the *ALK* transcript), and probe targeting human ALK exon 19 to exon 29 (corresponding to the 3′ region of the ALK transcript) were also obtained from Advanced Cell Diagnostics. RNA expression was detected using the RNAscope^®^ 2.5 High Definition (HD)-RED Assay according to the manufacturer's instructions (Advanced Cell Diagnostics). The images were acquired with an Olympus u-tv0.63xc microscope.

### Confirmation of patient *ALK* rearrangement status

Patient *ALK* rearrangement status was evaluated either by FISH (Abbott, Chicago, IL, USA), or IHC (Roche, Basel, Switzerland), by pathologists in the Department of Pathology at Fudan University Shanghai Cancer Center using standard protocols. FISH-positive cases were defined as those with two positive *ALK* rearrangement patterns. Positive cases were defined as more than 15% break-apart signals or isolated red signals in 50 tumor cells. Finally, FISH-negative cases were defined as those with overlapping red and green signals (yellowish) in tumor cells, which was indicative of no *ALK* rearrangement. IHC staining was scored by two pathologists as follows: 0 (no staining); 1 (faint, cytoplasmic staining); 2 (moderate, smooth cytoplasmic staining); 3 (intense, granular cytoplasmic staining) in more than 10% of the tumor cells. Anti-ALK IHC staining results were interpreted using a binary scoring system: positive (3+ or 2+) or negative (1+ or 0). *ALK* hybrid amplification-positive samples were subjected to sequencing analysis in order to determine the specific fusion partner.

## SUPPLEMENTARY MATERIALS FIGURES AND TABLES








