# Supplementary material for: Detection of ALK rearrangements in lung cancer patients using a homebrew PCR assay
Source: Oncotarget. 2016 Dec 10;8(5):7722–8. doi: 10.18632/oncotarget.13886 (PMC5352355; doi:10.18632/oncotarget.13886)
Supplement: Supplementary file 1 [file oncotarget-08-7722-s001.pdf]

Detection of *ALK* rearrangements in lung cancer patients using a homebrew PCR assay

Supplementary Materials

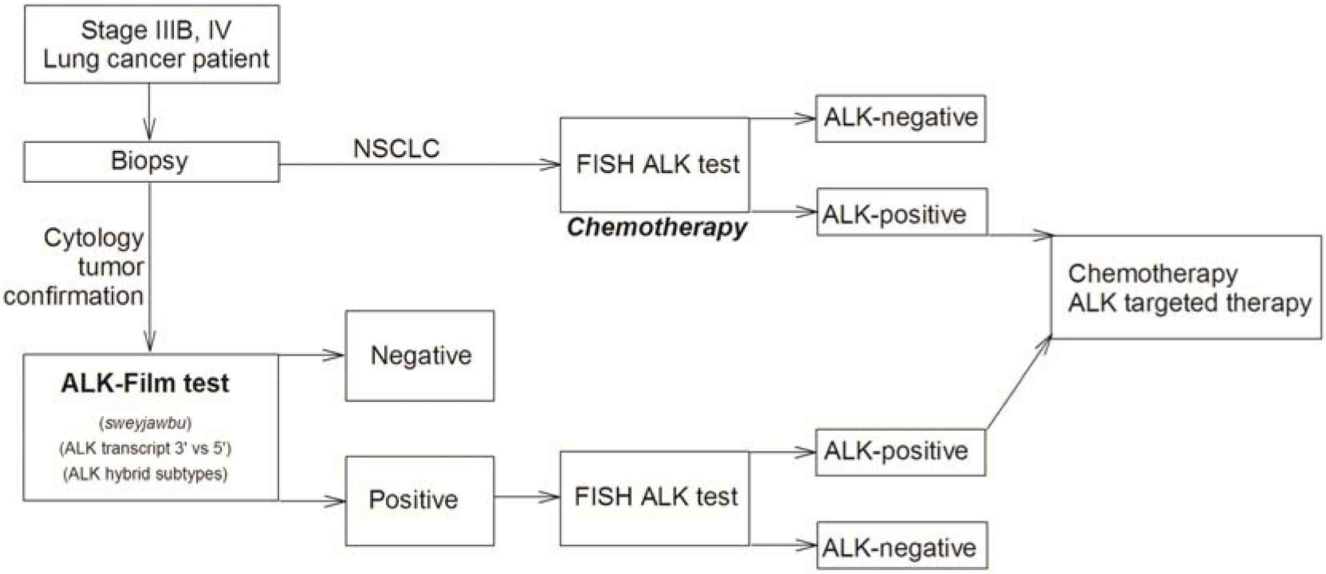

Supplementary Figure 1: Diagnostic screening algorithm for *ALK* rearrangement status. (**1**) Diagnostic screening algorithm to assess *ALK* rearrangement status using multiplex PCR on a FilmArray platform (ALK-Film test), and the FISH ALK test for confirmation in lung cancer patients. (**2**) Patients who present with advanced disease are diagnosed with NSCLC based on a pathological examination. These patients have a median untreated life expectancy of approximately 16 weeks. Therefore, they would prefer to start therapeutic treatment as early as possible. If a patient is eligible for targeted therapy, it could have better clinical efficacy and less severe side effects. However, current clinical practices at Fudan Univesity Shanghai Cancer Center take 7 working days to delivery final companion test results on FISH platfrom or IHC platform. (**3**) The FilmArray system is an CFDA-, FDA-, CE-IVD-, and TGA-certified multiplex PCR system that integrates sample preparation, amplification, detection, and analysis. It requires just a few minutes of hands-on-time and the turnaround time is approximately 1 hour. Faster results may in turn lead to better patient care. Samples from nasopharyngeal swabs could be applied to its respiratory panel, and 17 viruses and 3 bacteria which cause upper respiratory tract infections detected simultaneously with an overall sensitivity and specificity of 95% and 99%, respectively. (**4**) When an ALK-Film test on a FilmArray platform becomes available, patients could be prescreened with ALK-Film test at three different levels: (1) expression of the non-coding RNA *sweyjawbu*; (2) comparison of the expression of the 5′ and 3′ regions of the *ALK* transcript; and (3) expression of known *ALK* hybrid subtype variants. After disassociation with gentleMACS dissociator (Miltenyi Biotec), samples from fine needle biopsy could be applied to the FilmArray platform. In the meantime, samples could be subjected to cytological examination to confirm the presence of tumor cells and reduce the possibility of biopsy failure. Cases with positive signals would be analyzed by FISH to confirm *ALK* rearrangement positivity. Cases with no positive signals at all three levels would be considered ALK-negative and would not be subjected to FISH test. (**5**) FilmArray testing could substantially reduce the cost, time, and labor compared to FISH analysis. The addition of an ALK-Film diagnostic screening test could change current clinical practices and result in more effective treatment for *ALK* rearrangement-positive patients. This algorithm represents an accurate, efficient, economic, and convenient screening method to identify *ALK*-rearrangement positive NSCLC patients who will benefit from ALK-targeted therapy. The results can be confirmed by FISH. Note: NSCLC patients would also be subjected to testing for *EGFR* mutations, ROS1 rearrangement, which is not shown in this diagram.

Supplementary Table S1: Lung cancer patients' sample characteristics.
See Supplementary_Table_S1

Supplementary Table S2: Primer sequences used in current study.
See Supplementary_Table_S2

Supplementary Table S3: Top 20 differentially expressed genes

Probeset	ALK positive samples	ALK negative samples	Standard fold change
	Average probeset signal intensity		
242964_at	730.52	19.54	**24.18**
208212_s_at	1607.32	53.94	**11.34**
208211_s_at	114.94	20.02	**11.33**
242286_at	272.21	28.74	3.47
1562378_s_at	309.31	142.83	3.41
1570291_at	77.61	17.82	3.4
210753_s_at	78.34	34.13	3.24
1558388_a_at	77.2	12.6	3.23
241535_at	70.03	17.54	3.23
1556687_a_at	75.94	20.71	3.23
1558387_at	23.23	9.07	3.2
231384_at	254.66	19.74	3.13
223572_at	170.08	30.6	3.13
208470_s_at	11891.54	329.65	2.85
230593_at	29.07	14.72	2.81
227145_at	1293.2	301.36	2.77
1555675_at	79.3	23.8	2.74
216546_s_at	46.93	29.62	2.72
215976_at	54.11	23.39	2.71
211898_s_at	30.53	18.93	2.71

Compare 11 ALK positive tumors vs 235 ALK negative samples, from public microarray dataset E-GEOD-31210

Supplementary Table S4: Result table for lung cancer patients.
See Supplementary_Table_S4
