# Supplementary material for: Detection of ALK rearrangements in lung cancer patients using a homebrew PCR assay
Source: Oncotarget. 2016 Dec 10;8(5):7722–8. doi: 10.18632/oncotarget.13886 (PMC5352355; doi:10.18632/oncotarget.13886)
Supplement: Supplementary file 2 [file oncotarget-08-7722-s002.docx]

**Supplementary table 1: Lung cancer patients’ sample characteristics**

| **Sample** | **Age** | **Gender** | **Smoking** | **Tissue** | **Subtype** | **Grade of Diff.** | **T** | **N** | **M** | **Stage** |
| --- | --- | --- | --- | --- | --- | --- | --- | --- | --- | --- |
| **Patient 1** | 43 | Female | No | Lung tumor | ADC | Poorly | T1 | N1 | M0 | IIA |
| **Patient 2** | 61 | Female | No | Lung tumor | ADC | Poorly | T1 | N0 | M0 | IA |
| **Patient 3** | 57 | Female | No | Lung tumor | ADC | Poorly | T1 | Nx | M0 | IA |
| **Patient 4** | 55 | Female | No | Lung tumor | ADC | Poorly | T1 | N0 | M0 | IA |
| **Patient 5** | 39 | Female | No | Lung tumor | ADC | Moderately | T1 | N0 | M0 | IA |
| **Patient 6** | 58 | Male | NA | Lung tumor | LCC | NA | NA | NA | NA | NA |
| **Patient 7** | 59 | Male | Yes | Lung tumor | ADC | Moderately | T2 | N2 | M0 | IIIA |
| **Patient 8** | 60 | Female | No | Lung tumor | ADC | Moderately | T2 | N0 | M0 | IB |
| **Patient 9** | 64 | Male | Yes | Lung tumor | ADC | NA | T2 | N0 | M0 | IB |
| **Patient 10** | 37 | Female | No | Lung tumor | ADC | NA | T1 | N1 | M0 | IIB |
| **Patient 11** | 54 | Female | Yes | Lung tumor | ADC | Moderately | T1 | N2 | M0 | IIIA |
| **Patient 12** | 72 | Male | No | Lung tumor | ADC | Moderately | T1 | N0 | M0 | IA |
| **Patient 13** | 74 | Female | No | Lung tumor | ADC | Moderately | T1 | N0 | M0 | IA |
| **Patient 14** | 49 | Male | Yes | Lung tumor | ADC | Moderately | T2 | N2 | M0 | IIIB |
| **Patient 15** | 41 | Female | No | Lung tumor | ADC | Moderately | T1 | N0 | M0 | IA |
| **Patient 16** | 62 | Male | Yes | Lung tumor | ADC | Poorly | T1 | N1 | M0 | IIB |
| **Patient 17** | 70 | Male | Yes | Lung tumor | ADC | Moderately | T3 | N1 | M0 | IIIA |
| **Patient 18** | 53 | Female | No | Lung tumor | ADC | Poorly | T1 | N0 | M1 | IV |
| **Patient 19** | 56 | Female | No | Lung tumor | ADC | Moderately | T1 | N0 | M0 | IA |
| **Patient 20** | 43 | Female | No | Lung tumor | ADC | Poorly | T2 | N3 | M0 | IIIB |
| **Patient 21** | 56 | Male | Yes | Lung tumor | ADC | Poorly | T1 | N3 | M0 | IIIB |
| **Patient 22** | 56 | Female | No | Lung tumor | ADC | Poorly | T1 | N1 | M0 | IIB |
| **Patient 23** | 42 | Female | No | Lung tumor | ADC | Moderately | T1 | N2 | M0 | IIIA |
| **Patient 24** | 49 | Male | Yes | Lung tumor | ADC | Moderately | T2 | N1 | M0 | IIB |
| **Patient 25** | 51 | Female | No | Lung tumor | ADC | Moderately | T1 | N0 | M0 | IA |
| **Patient 26** | 51 | Female | No | Lung tumor | ADC | Moderately | T2 | N3 | M0 | IIIB |
| **Patient 27** | 63 | Female | No | Lung tumor | ADC | Poorly | T2 | N2 | M0 | IIIA |
| **Patient 28** | 47 | Male | Yes | Lung tumor | ADC | Moderately | T2 | N3 | M1 | IV |
| **Patient 29** | 64 | Female | No | Lung tumor | ADC | Moderately | T1 | N0 | M0 | IA |
| **Patient 30** | 54 | Female | No | Lung tumor | ADC | NA | T1 | N1 | M0 | IIB |
| **Patient 31** | 41 | Male | No | Lung tumor | ADC | Moderately | T1 | N0 | M0 | IA |
| **Patient 32** | 45 | Male | No | Lung tumor | ADC | Poorly | T1 | N2 | M0 | IIIA |
| Patient 33 | 69 | Male | Yes | Lung tumor | ADC | Poorly | T1 | N3 | M0 | IIIB |
| Patient 34 | 61 | Female | No | Lung tumor | ADC | Poorly | T1 | N0 | M0 | IA |
| Patient 35 | 60 | Male | No | Lung tumor | ADC | Moderately | T2 | N1 | M0 | IIB |
| Patient 36 | 65 | Male | Yes | Lung tumor | ADC | Moderately | T3 | N1 | M1 | IV |
| Patient 37 | 57 | Male | No | Lung tumor | ADC | Poorly | T2 | N0 | M0 | IIA |
| Patient 38 | 53 | Male | No | Lung tumor | ADC | Moderately | T1 | N0 | M0 | IA |
| Patient 39 | 57 | Male | Yes | Lung tumor | ADC | Moderately | T1 | N0 | M0 | IA |
| Patient 40 | 61 | Female | No | Lung tumor | ADC | Moderately | T1 | N0 | M0 | IA |
| Patient 41 | 65 | Male | No | Lung tumor | ADC | Moderately | T1 | Nx | M0 | IA |
| Patient 42 | 55 | Male | Yes | Lung tumor | ADC | Poorly | T2 | N1 | M0 | IIB |
| Patient 43 | 60 | Female | No | Lung tumor | ADC | Moderately | T1 | N1 | M0 | IIA |
| Patient 44 | 60 | Male | Yes | Lung tumor | ADC | Poorly | T2 | N0 | M0 | IIA |
| Patient 45 | 65 | Male | Yes | Lung tumor | ADC | Moderately | T1 | N0 | M0 | IA |
| Patient 46 | 56 | Male | No | Lung tumor | ADC | Moderately | T2 | N0 | M0 | IB |
| Patient 47 | 73 | Male | Yes | Lung tumor | ADC | Moderately | T2 | Nx | M0 | ？ |
| Patient 48 | 62 | Female | No | Lung tumor | ADC | Poorly | T1 | N0 | M0 | IA |
| Patient 49 | 60 | Female | No | Lung tumor | ADC | Poorly | T3 | N0 | M0 | IIB |
| Patient 50 | 42 | Male | Yes | Lung tumor | ADC | Poorly | T2 | N2 | M0 | IIIA |
| Patient 51 | 59 | Male | No | Lung tumor | ADC | Moderately | T2 | N1 | M0 | IIA |
| Patient 52 | 50 | Female | No | Lung tumor | ADC | Moderately | T1 | N0 | M0 | IA |
| Patient 53 | 57 | Male | No | Lung tumor | ADC | Moderately | T1 | N1 | M0 | IIA |
| Patient 54 | 50 | Female | No | Lung tumor | ADC | Well | T2 | N1 | M0 | IIB |
| Patient 55 | 57 | Female | No | Lung tumor | ADC | Moderately | T1 | N0 | M0 | IA |
| Patient 56 | 54 | Female | No | Lung tumor | ADC | Poorly | T2 | N0 | M1 | IV |
| Patient 57 | 76 | Male | Yes | Lung tumor | ADC | Moderately | T1 | N0 | M0 | IA |
| Patient 58 | 59 | Male | Yes | Lung tumor | ADC | Moderately | T1 | N2 | M0 | IIIA |
| Patient 59 | 58 | Male | Yes | Lung tumor | ADC | Poorly | T1 | N0 | M0 | IA |
| Patient 60 | 75 | Male | No | Lung tumor | ADC | Moderately | T1 | N2 | M0 | IIIA |
| Patient 61 | 62 | Male | Yes | Lung tumor | LCC | Poorly | T2 | N2 | M0 | IIIA |
| Patient 62 | 64 | Female | No | Lung tumor | LCC | Poorly | T3 | N1 | M0 | IIIA |
| Patient 63 | 58 | Male | Yes | Lung tumor | LCC | Poorly | T2 | Nx | M0 | IB |
| Patient 64 | 73 | Male | Yes | Lung tumor | SCC | Poorly | T2 | N0 | M0 | IB |
| Patient 65 | 65 | Male | Yes | Lung tumor | SCC | Poorly | T1 | N0 | M0 | IA |
| Patient 66 | 64 | Female | No | Lung tumor | SCC | Poorly | T2 | N1 | M0 | IIA |
| Patient 67 | 37 | Male | No | Lung tumor | SCC | Poorly | T2 | N0 | M0 | IB |
| Patient 68 | 56 | Male | Yes | Lung tumor | SCC | Moderately | T1 | N0 | M0 | IA |
| Patient 69 | 60 | Male | Yes | Lung tumor | SCC | Moderately | T2 | N1 | M0 | IIA |
| Patient 70 | 59 | Male | Yes | Lung tumor | SCC | Poorly | T2 | N0 | M0 | IB |
| Patient 71 | 70 | Male | Yes | Lung tumor | SCC | Poorly | T2 | N0 | M0 | IIA |
| Patient 72 | 67 | Female | Yes | Lung tumor | SCC | Poorly | T2 | N0 | M0 | IIA |
| Patient 73 | 55 | Male | No | Lung tumor | SCC | Poorly | T1 | N0 | M0 | IA |
| Patient 74 | 65 | Male | No | Lung tumor | SCC | Poorly | T2 | N0 | M0 | IIA |
| Patient 75 | 60 | Male | Yes | Lung tumor | SCC | Poorly | T2 | N0 | M0 | IB |
| Patient 76 | 49 | Male | Yes | Lung tumor | SCC | Poorly | T3 | N0 | M0 | IIB |
| Patient 77 | 63 | Male | Yes | Lung tumor | SCC | Poorly | T3 | Nx | M1 | IV |
| Patient 78 | 61 | Male | No | Lung tumor | SCC | Poorly | T1 | N0 | M0 | IA |
| Patient 79 | 76 | Male | No | Lung tumor | SCC | Poorly | T3 | N0 | M0 | IIB |
| Patient 80 | 60 | Male | Yes | Lung tumor | SCC | Poorly | T2 | N0 | M0 | IIA |
| Patient 81 | 68 | Male | Yes | Lung tumor | SCC | Moderately | T1 | N2 | M0 | IIIA |
| Patient 82 | 64 | Male | Yes | Lung tumor | SCC | Poorly | T2 | N0 | M0 | IIA |
| Patient 83 | 64 | Male | Yes | Lung tumor | SCC | Poorly | T3 | N0 | M0 | IIB |

- NA : data not available.
