# Supplementary material for: Detection of ALK rearrangements in lung cancer patients using a homebrew PCR assay
Source: Oncotarget. 2016 Dec 10;8(5):7722–8. doi: 10.18632/oncotarget.13886 (PMC5352355; doi:10.18632/oncotarget.13886)
Supplement: Supplementary file 3 [file oncotarget-08-7722-s003.docx]

**Supplementary table 2: Primer sequences used in current study**

| Template | 5' primer | 3' primer | References |
| --- | --- | --- | --- |
| EML4-ALK.E2;A20 | CATGTGGCCTCAGTGAAA | TGCTCAGCTTGTACTCAG | Clin Cancer Res 2008; 14: 6618-24 |
| EML4-ALK.E2;A20ins117 | CATGTGGCCTCAGTGAAA | TGCTCAGCTTGTACTCAG | Clin Cancer Res 2008; 14: 6618-24 |
| EML4-ALK.E3;A20ins53 | ACTCTTTCATCTGCTGCTA | TGCTCAGCTTGTACTCAG | J Thorac Oncol. 2013; 8: 883-91 |
| EML4-ALK.E6;A19 | AAGATGTCATCATCAACCAA | TGCTCAGCTTGTACTCAG | Clin Cancer Res. 2012; 18:4725-32 |
| EML4-ALK.E6;A20 | AAGATGTCATCATCAACCAA | TGCTCAGCTTGTACTCAG | Cancer Res 2008; 68: 4971-6, Cancer 2009; 115: 1723-33 |
| EML4-ALK.E6ins33;A20 | AAGATGTCATCATCAACCAA | TGCTCAGCTTGTACTCAG | Cancer Res 2008; 68: 4971-6 |
| EML4-ALK.E6;A20ins18 | AAGATGTCATCATCAACCAA | TGCTCAGCTTGTACTCAG | Int J Biomed Sci. 2012; 8: 1-6 |
| EML4-ALK.E13;A20 | CCTGGGAAAGGACCTAAA | TGCTCAGCTTGTACTCAG | Cancer 2009; 115: 1723-33, Lung Cancer. 2014; 84: 215-21, Mol Cancer Res. 2009; 7: 1466-76 |
| EML4-ALK.E13;A20ins69 | CCTGGGAAAGGACCTAAA | TGCTCAGCTTGTACTCAG | Clin Cancer Res 2009; 15: 3143-9 |
| EML4-ALK.E14;A20del12 | GGATGTTATTAACTGGAGGAG | TGCTCAGCTTGTACTCAG | Clin Cancer Res 2009; 15: 3143-9 |
| EML4-ALK.E14ins11;A20del49 | GGATGTTATTAACTGGAGGAG | TGCTCAGCTTGTACTCAG | Clin Cancer Res 2008; 14: 6618-24 |
| EML4-ALK.E17;A20ins33 | GTGGTGGCCATAGGAACG | TGCTCAGCTTGTACTCAG | Cancer Genet. 2011; 204: 45-52 |
| EML4-ALK.E17ins65;A20ins33 | GTGGTGGCCATAGGAACG | TGCTCAGCTTGTACTCAG | Cancer Genet. 2011; 204: 45-52 |
| EML4-ALK.E17ins68;A20 | GTGGTGGCCATAGGAACG | TGCTCAGCTTGTACTCAG | Ann Surg Oncol 2010; 17: 889-97 |
| EML4-ALK.E18;A20 | GTGGTGGCCATAGGAACG | TGCTCAGCTTGTACTCAG | Cancer 2009; 115: 1723-33 |
| EML4-ALK.E19;A20del14 | TGGAAGAAAATATAGCAGATATGG | TGCTCAGCTTGTACTCAG | Lung Cancer. 2014; 84: 215-21 |
| EML4-ALK.E20;A20 | ATATAATGTCTAACTCGGGAGA | TGCTCAGCTTGTACTCAG | Cancer 2009; 115: 1723-33, Mol Cancer Res. 2009; 7: 1466-76, Ann Surg Oncol 2010; 17: 889-97 |
| EML4-ALK.E20;A20ins18 | ATATAATGTCTAACTCGGGAGA | TGCTCAGCTTGTACTCAG | Ann Surg Oncol 2010; 17: 889-97 |
| EML4-ALK.E21;A20 | TATTGTACTGGGACATTCCA | TGCTCAGCTTGTACTCAG | Mol Cancer Res. 2009; 7: 1466-76 |
| HIP1-ALK.H21;A20 | GGACTTCTCCATTCCATAAC | TGCTCAGCTTGTACTCAG | J Thorac Oncol. 2014; 9: 419-22 |
| KIF5B-ALK.K15;A20del14 | AAAGACCTTGCAGAAATAGG | TGCTCAGCTTGTACTCAG | Cancer. 2011; 117: 2709-18 |
| KIF5B-ALK.K17;A20 | ATGTCAGCTTCGTATCTCT | TGCTCAGCTTGTACTCAG | Clin Cancer Res. 2012; 18:4725-32 |
| KIF5B-ALK.K24;A20 | AAGAAGTAGATCGCATAAAGG | TGCTCAGCTTGTACTCAG | Clin Cancer Res. 2009; 15: 3143-9 |
| KLC1-ALK.K9;A20 | ATGAAAGGGAGTTTGGTTCT | TGCTCAGCTTGTACTCAG | PLoS One. 2012; 7: e31323 |
| TFG-ALK.T4;A20 | ATTGGATAGCTTGGAACC | TGCTCAGCTTGTACTCAG | Cell. 2007; 131: 1190-203 |
| TPR-ALK.T15;A20 | AGCGTGATATGTACCGTAT | TGCTCAGCTTGTACTCAG | J Thorac Oncol. 2014; 9: 563-6 |
| STRN-ALK.S3;A20 | GAGATATGAAGCCTCCAAG | TGCTCAGCTTGTACTCAG | J Pathol. 2013; 230: 270-6 |
| ALK 5' transcript (exon 18) | GGGAAGTGAATATTAAGCATTATC | AGAAGCAGATGACCTTGT | Clin Cancer Res. 2012; 18:4725-32 |
| ALK 3' transcript (exon 24-25) | CCTGTGGCTGTCAGTATT | AGGTCAAGAGGCAGTTTC | Clin Cancer Res. 2012; 18:4725-32 |
| *sweyjawbu* | CCTAAAGGATACTGACAGAGGAGTTG | GTGRTAGAAAGGCACAACCTGAAA | - |
| HPRT1 | ACAAAGCCTAAGATGAGAG | GCCACAGAACTAGAACAT | Acta Biochim Pol. 2009; 56(2): 307-16 |
| ESD | TTACCACCAAAGGCAGAA | GAAGCAGACTGATGATAACC | Acta Biochim Pol. 2009; 56(2): 307-16 |
