# Supplementary material for: Detection of ALK rearrangements in lung cancer patients using a homebrew PCR assay
Source: Oncotarget. 2016 Dec 10;8(5):7722–8. doi: 10.18632/oncotarget.13886 (PMC5352355; doi:10.18632/oncotarget.13886)
Supplement: Supplementary file 4 [file oncotarget-08-7722-s004.docx]

**Supplementary table 4: Result table for lung cancer patients**

| Numbers | Tissue | ALK fusion status | | | |
| --- | --- | --- | --- | --- | --- |
|  |  | Homebrew PCR | FISH | IHC | Fusion subtype |
| **Patient 1** | ADC tumor | + | Positive (~ 50%) | No data | EML4-ALK (E13;A20) |
| **Patient 2** | ADC tumor | + | Positive (NA) | No data | EML4-ALK (E3;A20) |
| **Patient 3** | ADC tumor | + | Positive (~ 50%) | No data | EML4-ALK (E6;A20) |
| **Patient 4** | ADC tumor | + | Positive (~ 40%) | Positive | EML4-ALK (E13;A20) |
| **Patient 5** | ADC tumor | + | Positive (~ 25%) | Positive | EML4-ALK (E6;A20) |
| **Patient 6** | LCC tumor | + | No data | No data | EML4-ALK (E6;A20) |
| **Patient 7** | ADC tumor | + | No data | Positive | EML4-ALK (E13;A20) |
| **Patient 8** | ADC tumor | + | No data | Positive | KIF5B-ALK (K15;A20) |
| **Patient 9** | ADC tumor | + | No data | Positive | EML4-ALK (E13;A20) |
| **Patient 10** | ADC tumor | + | No data | Positive | EML4-ALK (E18;A20) |
| **Patient 11** | ADC tumor | + | No data | Positive | EML4-ALK (E6;A20) |
| **Patient 12** | ADC tumor | + | No data | Positive | EML4-ALK (E13;A20) |
| **Patient 13** | ADC tumor | + | No data | Positive | EML4-ALK (E6;A20) |
| **Patient 14** | ADC tumor | + | No data | Positive | EML4-ALK (E6;A20) |
| **Patient 15** | ADC tumor | + | No data | Positive | EML4-ALK (E6;A20) |
| **Patient 16** | ADC tumor | + | No data | Positive | EML4-ALK (E13;A20) |
| **Patient 17** | ADC tumor | + | No data | Positive | EML4-ALK (E13;A20) |
| **Patient 18** | ADC tumor | + | No data | Positive | EML4-ALK (E20;A20) |
| **Patient 19** | ADC tumor | + | No data | Positive | EML4-ALK (E20;A20) |
| **Patient 20** | ADC tumor | + | No data | Positive | EML4-ALK (E13;A20) |
| **Patient 21** | ADC tumor | + | No data | Positive | EML4-ALK (E13;A20) |
| **Patient 22** | ADC tumor | + | No data | Positive | EML4-ALK (E13;A20) |
| **Patient 23** | ADC tumor | + | No data | Positive | EML4-ALK (E6;A20) |
| **Patient 24** | ADC tumor | + | No data | Positive | EML4-ALK (E6;A20) |
| **Patient 25** | ADC tumor | + | No data | Positive | EML4-ALK (E20;A20) |
| **Patient 26** | ADC tumor | + | No data | Positive | EML4-ALK (E6;A20) |
| **Patient 27** | ADC tumor | + | No data | Positive | EML4-ALK (E20;A20) |
| **Patient 28** | ADC tumor | + | No data | Positive | EML4-ALK (E6;A20) |
| **Patient 29** | ADC tumor | + | No data | Positive | EML4-ALK (E6;A20) |
| **Patient 30** | ADC tumor | + | No data | Positive | EML4-ALK (E13;A20) |
| **Patient 31** | ADC tumor | + | No data | Positive | EML4-ALK (E13;A20) |
| **Patient 32** | ADC tumor | + | No data | Positive | EML4-ALK (E13;A20) |
| Patient 33 | ADC tumor | - | No data | Negative | No data |
| Patient 34 | ADC tumor | - | No data | Negative | No data |
| Patient 35 | ADC tumor | - | No data | Negative | No data |
| Patient 36 | ADC tumor | - | Negative (~ 3%) | No data | EML4-ALK (E6;A20) |
| Patient 37 | ADC tumor | - | Negative (~ 0%) | No data | No data |
| Patient 38 | ADC tumor | - | No data | Negative | No data |
| Patient 39 | ADC tumor | - | No data | No data | No data |
| Patient 40 | ADC tumor | - | No data | Negative | No data |
| Patient 41 | ADC tumor | - | No data | No data | No data |
| Patient 42 | ADC tumor | - | No data | No data | No data |
| Patient 43 | ADC tumor | - | No data | Negative | No data |
| Patient 44 | ADC tumor | - | No data | No data | No data |
| Patient 45 | ADC tumor | - | No data | Negative | No data |
| Patient 46 | ADC tumor | - | No data | Negative | No data |
| Patient 47 | ADC tumor | - | No data | Negative | No data |
| Patient 48 | ADC tumor | - | No data | Negative | No data |
| Patient 49 | ADC tumor | - | No data | Negative | No data |
| Patient 50 | ADC tumor | - | No data | No data | No data |
| Patient 51 | ADC tumor | - | No data | No data | No data |
| Patient 52 | ADC tumor | - | No data | Negative | No data |
| Patient 53 | ADC tumor | - | No data | Negative | No data |
| Patient 54 | ADC tumor | - | No data | No data | No data |
| Patient 55 | ADC tumor | - | No data | No data | No data |
| Patient 56 | ADC tumor | - | No data | Negative | No data |
| Patient 57 | ADC tumor | - | No data | Negative | No data |
| Patient 58 | ADC tumor | - | No data | Negative | No data |
| Patient 59 | ADC tumor | - | No data | No data | No data |
| Patient 60 | ADC tumor | - | No data | No data | No data |
| Patient 61 | LCC tumor | - | Negative (~ 0%) | Negative | No data |
| Patient 62 | LCC tumor | - | No data | No data | No data |
| Patient 63 | LCC tumor | - | No data | No data | No data |
| Patient 64 | SCC tumor | - | No data | Negative | No data |
| Patient 65 | SCC tumor | - | No data | Negative | No data |
| Patient 66 | SCC tumor | - | No data | Negative | No data |
| Patient 67 | SCC tumor | - | No data | Negative | No data |
| Patient 68 | SCC tumor | - | No data | Negative | No data |
| Patient 69 | SCC tumor | - | No data | Negative | No data |
| Patient 70 | SCC tumor | - | No data | Negative | No data |
| Patient 71 | SCC tumor | - | No data | Negative | No data |
| Patient 72 | SCC tumor | - | No data | Negative | No data |
| Patient 73 | SCC tumor | - | No data | Negative | No data |
| Patient 74 | SCC tumor | - | No data | Negative | No data |
| Patient 75 | SCC tumor | - | No data | Negative | No data |
| Patient 76 | SCC tumor | - | No data | Negative | No data |
| Patient 77 | SCC tumor | - | No data | No data | No data |
| Patient 78 | SCC tumor | - | No data | Negative | No data |
| Patient 79 | SCC tumor | - | No data | Negative | No data |
| Patient 80 | SCC tumor | - | No data | Negative | No data |
| Patient 81 | SCC tumor | - | No data | No data | No data |
| Patient 82 | SCC tumor | - | No data | No data | No data |
| Patient 83 | SCC tumor | - | No data | Negative | No data |

- ADC: adenocarcinoma; LCC: Large cell carcinoma; SCC: Squamous cell carcinoma; No amplification: No amplification detected after 40 cycles PCR.
